# 7-Chloro-4-(2-hy­droxy­ethyl­amino)­quinolin-1-ium chloride

**DOI:** 10.1107/S1600536814004565

**Published:** 2014-03-05

**Authors:** Ivson L. Gama, Marcus V. N. de Souza, James L. Wardell, Edward R. T. Tiekink

**Affiliations:** a Fundação Oswaldo Cruz, Instituto de Tecnologia em Fármacos–Farmanguinhos, R. Sizenando Nabuco, 100, Manguinhos, 21041-250, Rio de Janeiro, RJ, Brazil; b Chemistry Department, University of Aberdeen, Old Aberdeen, AB24 3UE, Scotland; c Department of Chemistry, University of Malaya, 50603 Kuala Lumpur, Malaysia

## Abstract

In the title salt, C_11_H_12_ClN_2_O^+^·Cl^−^, the ten non-H atoms comprising the quinolinium residue are coplanar (r.m.s. deviation = 0.041 Å) and the hy­droxy­ethyl group is approximately perpendicular to this plane [C_ring_—N—C_methyl­ene_—C torsion angle = −74.61 (18)°]. A supra­molecular chain aligned along [101] mediated by charge-assisted O/N—H⋯Cl^−^ hydrogen bonds features in the crystal packing. Chains are connected into a three-dimensional architecture by C—H⋯O(hy­droxy) inter­actions.

## Related literature   

For the wide range of pharmacological activities of synthetic and natural products containing the quinoline nucleus, see: Andrade *et al.* (2007 ▶); Cunico *et al.* (2006 ▶); Font *et al.* (1997 ▶); Kaminsky & Meltzer (1968 ▶); Musiol *et al.* (2006 ▶); Nakamura *et al.* (1999 ▶); Sloboda *et al.*, (1991 ▶); de Souza *et al.* (2014 ▶); Tanenbaum & Tuffanelli (1980 ▶); Warshakoon *et al.* (2006 ▶). For the crystal structures of related 4-*R*N(H)-7-chloro­quinolines, see: Kaiser *et al.*, (2009 ▶).
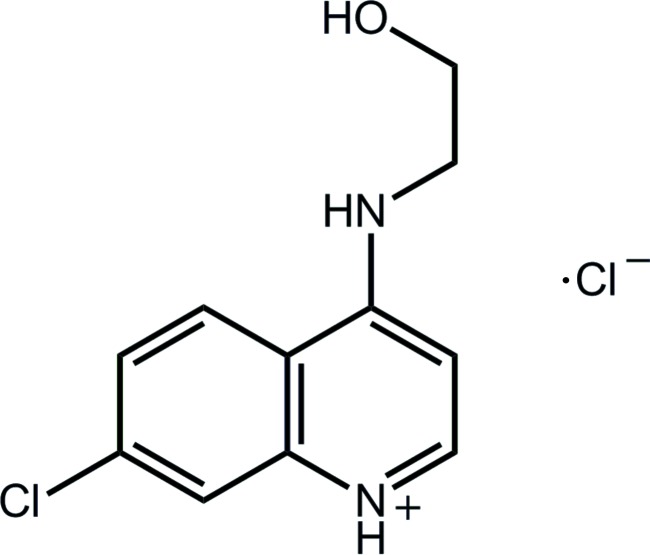


## Experimental   

### 

#### Crystal data  

C_11_H_12_ClN_2_O^+^·Cl^−^
*M* *_r_* = 259.13
Monoclinic, 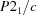

*a* = 8.2438 (13) Å
*b* = 16.405 (2) Å
*c* = 8.8561 (14) Å
β = 110.705 (2)°
*V* = 1120.3 (3) Å^3^
*Z* = 4
Mo *K*α radiation
μ = 0.56 mm^−1^
*T* = 100 K
0.20 × 0.07 × 0.04 mm

#### Data collection  

Rigaku R-AXIS conversion diffractometer
Absorption correction: multi-scan (*CrystalClear-SM Expert*; Rigaku, 2013 ▶) *T*_min_ = 0.831, *T*_max_ = 1.000
7784 measured reflections
2581 independent reflections
2162 reflections with *I* > 2σ(*I*)
*R*_int_ = 0.036

#### Refinement  

*R*[*F* ^2^ > 2σ(*F* ^2^)] = 0.030
*wR*(*F* ^2^) = 0.076
*S* = 1.07
2581 reflections
154 parameters
3 restraints
H atoms treated by a mixture of independent and constrained refinement
Δρ_max_ = 0.37 e Å^−3^
Δρ_min_ = −0.24 e Å^−3^

### 

Data collection: *CrystalClear-SM Expert* (Rigaku, 2013 ▶); cell refinement: *CrystalClear-SM Expert*; data reduction: *CrystalClear-SM Expert*; program(s) used to solve structure: *SHELXS97* (Sheldrick, 2008 ▶); program(s) used to refine structure: *SHELXL97* (Sheldrick, 2008 ▶); molecular graphics: *ORTEP-3 for Windows* (Farrugia, 2012 ▶) and *DIAMOND* (Brandenburg, 2006 ▶); software used to prepare material for publication: *publCIF* (Westrip, 2010 ▶).

## Supplementary Material

Crystal structure: contains datablock(s) general, I. DOI: 10.1107/S1600536814004565/hg5387sup1.cif

Structure factors: contains datablock(s) I. DOI: 10.1107/S1600536814004565/hg5387Isup2.hkl

Supporting information file. DOI: 10.1107/S1600536814004565/hg5387Isup3.cml

CCDC reference: 988939

Additional supporting information:  crystallographic information; 3D view; checkCIF report

## Figures and Tables

**Table 1:** Hydrogen-bond geometry (Å, °)

*D*—H⋯*A*	*D*—H	H⋯*A*	*D*⋯*A*	*D*—H⋯*A*
O1—H1o⋯Cl2	0.84 (2)	2.30 (2)	3.1338 (14)	179 (2)
N1—H1n⋯Cl2^i^	0.88 (1)	2.29 (1)	3.1602 (15)	168 (1)
N2—H2n⋯Cl2^ii^	0.87 (1)	2.49 (2)	3.2949 (14)	154 (2)
C2—H2⋯O1^iii^	0.95	2.60	3.545 (2)	173

Symmetry codes: (i) 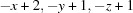; (ii) 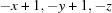; (iii) 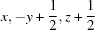.

**Fig. 1.**
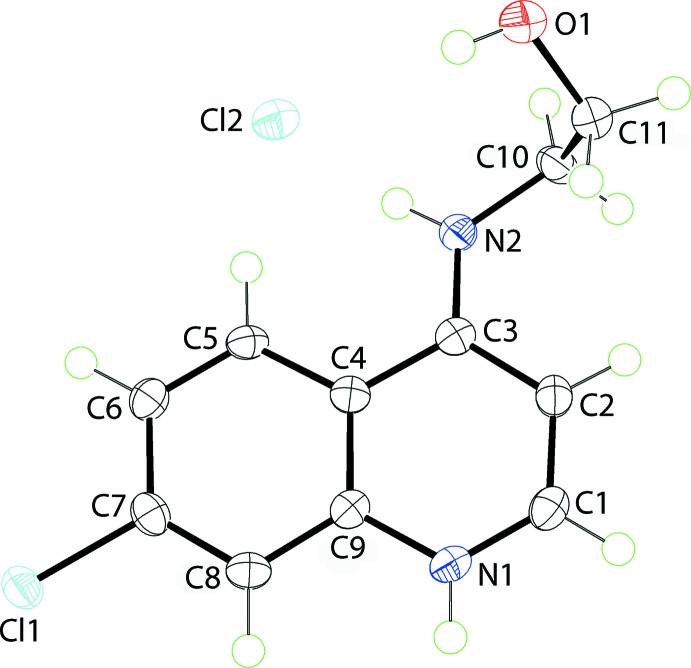
The molecular structures of the ions in (I) showing the atom-labelling scheme and displacement ellipsoids at the 70% probability level.

**Fig. 2.**
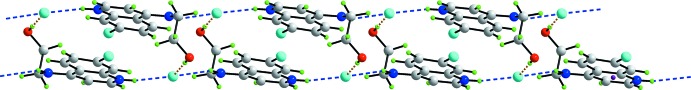
A view of the supramolecular chain along [1 0 1] in (I) showing O—H···Cl- and N—H···Cl- hydrogen bonds as orange and blue dashed lines, respectively.

**Fig. 3.**
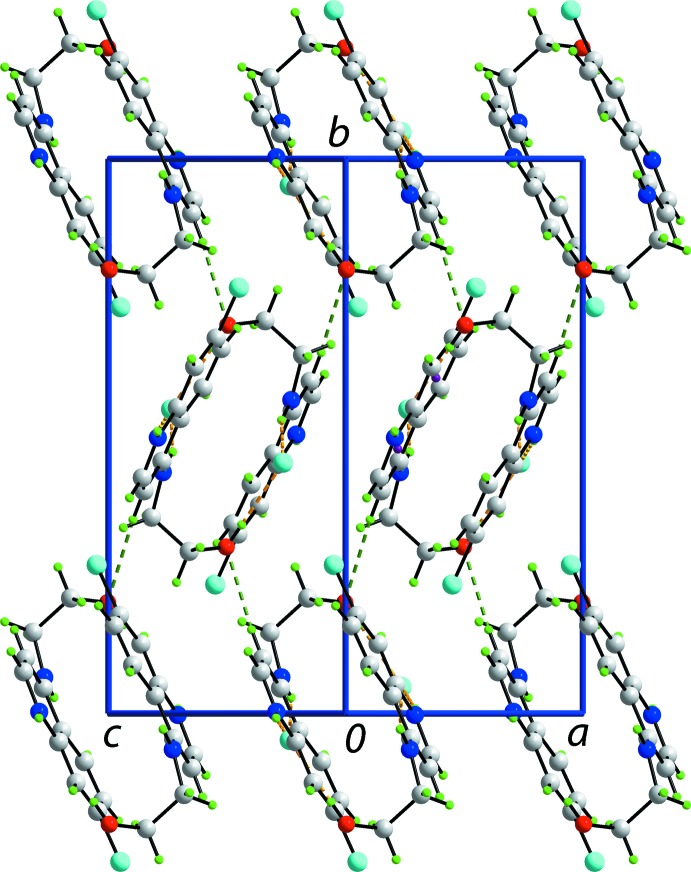
A view in projection down [1 0 1], the direction of the chain shown in Fig. 2, of the unit-cell contents of (I). The O—H···Cl-, N—H···Cl- and C—H···O interactions are shown as orange, blue and green dashed lines, respectively.

## supplementary crystallographic information

### 2.1. Synthesis and crystallization

A solution of 7-chloro-4-(2-hy­droxy­ethyl­amino)­quinoline (1 mmol) and FeCl_3_.6H_2_O (1 mmol) in EtOH (25 ml) was refluxed for 30 min. On leaving the reaction mixture at room temperature, crystals of the title compound were formed, M.pt: 538–541 K (dec.).

### 3. Refinement

Intensity data was collected at the National Crystallographic Service, England (Coles & Gale, 2012). The C-bound H atoms were geometrically placed (C—H = 0.95–0.99 Å) and refined as riding with *U*_iso_(H) = 1.2*U*_eq_(C). The O- and N-bound H atoms were located from a difference map and refined with O—H = 0.84±0.01 Å and N—H = 0.88±0.01 Å, respectively, and with *U*_iso_(H) = 1.5*U*_eq_(O) and 1.2*U*_eq_(N), respectively.

### 4. Results and discussion

The quinoline nucleus is found in many synthetic and natural products having a wide range of pharmacological activities, such as anti-viral (Font *et al.*, 1997), anti-cancer (Nakamura *et al.*, 1999; de Souza *et al.*, 2014), anti-bacterial (Kaminsky & Meltzer, 1968), anti-malarial (Tanenbaum & Tuffanelli, 1980; Cunico *et al.*, 2006; Andrade *et al.*, 2007), anti-fungal (Musiol *et al.*, 2006), anti-obesity (Warshakoon *et al.*, 2006) and anti-inflammatory (Sloboda *et al.*, 1991) activities. The crystal structures of a series of 4-RN(H)-7-chloro-quinolines was recently reported (Kaiser *et al.*, 2009). We now wish to report the crystal structure of the HCl salt of 4-(HOCH_2_CH_2_NH-7-chloro-quinoline, (I), obtained serendipiously from an attempted reaction to generate an iron complex.

The components of salt (I) are illustrated in Fig. 1. The 10 non-hydrogen atoms comprising the quinolinium residue are co-planar with a r.m.s. deviation of 0.041 Å. The hy­droxy­ethyl group is almost perpendicular to this plane as seen in the C3—N2—C10—C11 torsion angle of -74.61 (18)°.

In the crystal structure, charge-assisted O, N—H···Cl^-^ hydrogen bonds, Table 1, lead to a supra­molecular chain aligned along [1 0 1], Fig. 2. These are connected into a three-dimensional architecture by methyl­ene-C—H···O(hydroxyl) inter­actions, Fig. 3 & Table 1.

### Crystal data

**Table:**

C_11_H_12_ClN_2_O^+^·Cl^−^	*F*(000) = 536
*M**_r_* = 259.13	*D*_x_ = 1.536 Mg m^−^^3^
Monoclinic, *P*2_1_/*c*	Mo *K*α radiation, λ = 0.71075 Å
Hall symbol: -P 2ybc	Cell parameters from 14670 reflections
*a* = 8.2438 (13) Å	θ = 3.0–27.5°
*b* = 16.405 (2) Å	µ = 0.56 mm^−^^1^
*c* = 8.8561 (14) Å	*T* = 100 K
β = 110.705 (2)°	Prism, colourless
*V* = 1120.3 (3) Å^3^	0.20 × 0.07 × 0.04 mm
*Z* = 4	

### Data collection

**Table:**

Rigaku R-AXIS conversion diffractometer	2581 independent reflections
Radiation source: Sealed Tube	2162 reflections with *I* > 2σ(*I*)
Graphite monochromator	*R*_int_ = 0.036
Detector resolution: 10.0000 pixels mm^-1^	θ_max_ = 27.5°, θ_min_ = 2.5°
profile data from ω–scans	*h* = −10→9
Absorption correction: multi-scan (*CrystalClear*-SM Expert; Rigaku, 2013)	*k* = −20→21
*T*_min_ = 0.831, *T*_max_ = 1.000	*l* = −11→11
7784 measured reflections	

### Refinement

**Table:**

Refinement on *F*^2^	Primary atom site location: structure-invariant direct methods
Least-squares matrix: full	Secondary atom site location: difference Fourier map
*R*[*F*^2^ > 2σ(*F*^2^)] = 0.030	Hydrogen site location: inferred from neighbouring sites
*wR*(*F*^2^) = 0.076	H atoms treated by a mixture of independent and constrained refinement
*S* = 1.07	*w* = 1/[σ^2^(*F*_o_^2^) + (0.0353*P*)^2^ + 0.170*P*] where *P* = (*F*_o_^2^ + 2*F*_c_^2^)/3
2581 reflections	(Δ/σ)_max_ = 0.001
154 parameters	Δρ_max_ = 0.37 e Å^−^^3^
3 restraints	Δρ_min_ = −0.24 e Å^−^^3^

### Special details

**Table:**

Geometry. All s.u.'s (except the s.u. in the dihedral angle between two l.s. planes) are estimated using the full covariance matrix. The cell s.u.'s are taken into account individually in the estimation of s.u.'s in distances, angles and torsion angles; correlations between s.u.'s in cell parameters are only used when they are defined by crystal symmetry. An approximate (isotropic) treatment of cell s.u.'s is used for estimating s.u.'s involving l.s. planes.
Refinement. Refinement of *F*^2^ against ALL reflections. The weighted *R*-factor *wR* and goodness of fit *S* are based on *F*^2^, conventional *R*-factors *R* are based on *F*, with *F* set to zero for negative *F*^2^. The threshold expression of *F*^2^ > 2σ(*F*^2^) is used only for calculating *R*-factors(gt) *etc*. and is not relevant to the choice of reflections for refinement. *R*-factors based on *F*^2^ are statistically about twice as large as those based on *F*, and *R*- factors based on ALL data will be even larger.

### Fractional atomic coordinates and isotropic or equivalent isotropic displacement parameters (Å^2^)

**Table:**

	*x*	*y*	*z*	*U*_iso_*/*U*_eq_	
Cl1	1.02274 (5)	0.76756 (2)	0.47284 (4)	0.01888 (11)	
O1	0.53371 (15)	0.30013 (7)	0.03702 (13)	0.0206 (3)	
H1O	0.595 (2)	0.3400 (9)	0.033 (2)	0.031*	
N1	0.90024 (17)	0.49718 (8)	0.69236 (15)	0.0153 (3)	
H1N	0.9879 (17)	0.5083 (11)	0.7812 (14)	0.018*	
N2	0.50113 (17)	0.43490 (8)	0.27207 (15)	0.0153 (3)	
H2N	0.466 (2)	0.4713 (9)	0.1961 (17)	0.018*	
C1	0.8212 (2)	0.42491 (10)	0.67877 (18)	0.0162 (3)	
H1	0.8571	0.3886	0.7681	0.019*	
C2	0.6904 (2)	0.40138 (9)	0.54089 (18)	0.0152 (3)	
H2	0.6387	0.3492	0.5354	0.018*	
C3	0.63187 (19)	0.45441 (9)	0.40638 (17)	0.0131 (3)	
C4	0.72227 (19)	0.53172 (9)	0.41947 (18)	0.0135 (3)	
C5	0.68800 (19)	0.58698 (9)	0.28956 (17)	0.0145 (3)	
H5	0.5992	0.5747	0.1895	0.017*	
C6	0.7804 (2)	0.65795 (9)	0.30511 (18)	0.0157 (3)	
H6	0.7581	0.6940	0.2160	0.019*	
C7	0.90831 (19)	0.67665 (9)	0.45450 (18)	0.0154 (3)	
C8	0.94774 (19)	0.62514 (9)	0.58444 (18)	0.0153 (3)	
H8	1.0349	0.6389	0.6845	0.018*	
C9	0.85580 (19)	0.55131 (9)	0.56571 (17)	0.0133 (3)	
C10	0.4232 (2)	0.35374 (9)	0.24153 (19)	0.0172 (3)	
H10A	0.3101	0.3569	0.1517	0.021*	
H10B	0.4014	0.3348	0.3388	0.021*	
C11	0.5388 (2)	0.29206 (10)	0.19883 (18)	0.0177 (3)	
H11A	0.6598	0.2992	0.2736	0.021*	
H11B	0.5014	0.2363	0.2144	0.021*	
Cl2	0.75804 (5)	0.45204 (2)	0.02211 (4)	0.01726 (10)	

### Atomic displacement parameters (Å^2^)

**Table:**

	*U*^11^	*U*^22^	*U*^33^	*U*^12^	*U*^13^	*U*^23^
Cl1	0.0192 (2)	0.01435 (19)	0.02016 (19)	−0.00294 (15)	0.00339 (15)	0.00041 (15)
O1	0.0244 (6)	0.0209 (6)	0.0164 (5)	−0.0031 (5)	0.0071 (5)	−0.0019 (5)
N1	0.0159 (6)	0.0175 (7)	0.0112 (6)	0.0010 (5)	0.0030 (5)	0.0010 (5)
N2	0.0163 (6)	0.0137 (7)	0.0140 (6)	0.0000 (5)	0.0029 (5)	0.0005 (5)
C1	0.0194 (8)	0.0160 (7)	0.0146 (7)	0.0032 (6)	0.0079 (6)	0.0021 (6)
C2	0.0175 (7)	0.0133 (7)	0.0162 (7)	0.0000 (6)	0.0077 (6)	0.0008 (6)
C3	0.0130 (7)	0.0139 (7)	0.0138 (7)	0.0031 (6)	0.0064 (6)	−0.0010 (6)
C4	0.0133 (7)	0.0139 (7)	0.0141 (7)	0.0014 (6)	0.0058 (6)	−0.0016 (6)
C5	0.0143 (7)	0.0151 (7)	0.0120 (7)	0.0021 (6)	0.0021 (6)	−0.0012 (6)
C6	0.0160 (7)	0.0148 (7)	0.0158 (7)	0.0029 (6)	0.0050 (6)	0.0025 (6)
C7	0.0152 (7)	0.0126 (7)	0.0196 (7)	−0.0003 (6)	0.0078 (6)	−0.0016 (6)
C8	0.0133 (7)	0.0173 (8)	0.0143 (7)	0.0011 (6)	0.0035 (6)	−0.0021 (6)
C9	0.0144 (7)	0.0133 (7)	0.0131 (7)	0.0035 (6)	0.0060 (6)	0.0000 (6)
C10	0.0177 (8)	0.0156 (8)	0.0177 (7)	−0.0043 (6)	0.0053 (6)	−0.0034 (6)
C11	0.0209 (8)	0.0159 (8)	0.0164 (7)	−0.0023 (6)	0.0066 (6)	−0.0002 (6)
Cl2	0.01711 (19)	0.0195 (2)	0.01341 (17)	0.00029 (15)	0.00320 (14)	−0.00020 (14)

### Geometric parameters (Å, º)

**Table:**

Cl1—C7	1.7413 (16)	C4—C9	1.409 (2)
O1—C11	1.4250 (18)	C4—C5	1.413 (2)
O1—H1O	0.838 (9)	C5—C6	1.371 (2)
N1—C1	1.338 (2)	C5—H5	0.9500
N1—C9	1.3749 (19)	C6—C7	1.404 (2)
N1—H1N	0.879 (9)	C6—H6	0.9500
N2—C3	1.331 (2)	C7—C8	1.371 (2)
N2—C10	1.4615 (19)	C8—C9	1.407 (2)
N2—H2N	0.869 (9)	C8—H8	0.9500
C1—C2	1.368 (2)	C10—C11	1.526 (2)
C1—H1	0.9500	C10—H10A	0.9900
C2—C3	1.415 (2)	C10—H10B	0.9900
C2—H2	0.9500	C11—H11A	0.9900
C3—C4	1.455 (2)	C11—H11B	0.9900
			
C11—O1—H1O	108.4 (14)	C5—C6—H6	120.5
C1—N1—C9	121.21 (13)	C7—C6—H6	120.5
C1—N1—H1N	119.0 (12)	C8—C7—C6	122.19 (14)
C9—N1—H1N	119.6 (12)	C8—C7—Cl1	119.32 (12)
C3—N2—C10	123.26 (13)	C6—C7—Cl1	118.48 (12)
C3—N2—H2N	117.9 (12)	C7—C8—C9	118.27 (14)
C10—N2—H2N	118.6 (12)	C7—C8—H8	120.9
N1—C1—C2	122.38 (14)	C9—C8—H8	120.9
N1—C1—H1	118.8	N1—C9—C8	118.87 (13)
C2—C1—H1	118.8	N1—C9—C4	119.93 (14)
C1—C2—C3	120.30 (14)	C8—C9—C4	121.20 (14)
C1—C2—H2	119.8	N2—C10—C11	112.19 (13)
C3—C2—H2	119.8	N2—C10—H10A	109.2
N2—C3—C2	122.13 (14)	C11—C10—H10A	109.2
N2—C3—C4	120.72 (13)	N2—C10—H10B	109.2
C2—C3—C4	117.15 (13)	C11—C10—H10B	109.2
C9—C4—C5	117.95 (14)	H10A—C10—H10B	107.9
C9—C4—C3	118.95 (13)	O1—C11—C10	112.87 (13)
C5—C4—C3	123.05 (14)	O1—C11—H11A	109.0
C6—C5—C4	121.27 (14)	C10—C11—H11A	109.0
C6—C5—H5	119.4	O1—C11—H11B	109.0
C4—C5—H5	119.4	C10—C11—H11B	109.0
C5—C6—C7	119.06 (14)	H11A—C11—H11B	107.8
			
C9—N1—C1—C2	−1.4 (2)	C5—C6—C7—Cl1	−179.28 (11)
N1—C1—C2—C3	−0.9 (2)	C6—C7—C8—C9	0.0 (2)
C10—N2—C3—C2	−9.1 (2)	Cl1—C7—C8—C9	−178.78 (11)
C10—N2—C3—C4	170.11 (13)	C1—N1—C9—C8	−177.54 (14)
C1—C2—C3—N2	−177.69 (14)	C1—N1—C9—C4	1.4 (2)
C1—C2—C3—C4	3.0 (2)	C7—C8—C9—N1	176.63 (13)
N2—C3—C4—C9	177.74 (13)	C7—C8—C9—C4	−2.3 (2)
C2—C3—C4—C9	−3.0 (2)	C5—C4—C9—N1	−176.31 (13)
N2—C3—C4—C5	−5.2 (2)	C3—C4—C9—N1	0.9 (2)
C2—C3—C4—C5	174.04 (13)	C5—C4—C9—C8	2.6 (2)
C9—C4—C5—C6	−0.6 (2)	C3—C4—C9—C8	179.74 (13)
C3—C4—C5—C6	−177.64 (14)	C3—N2—C10—C11	−74.61 (18)
C4—C5—C6—C7	−1.6 (2)	N2—C10—C11—O1	−77.07 (16)
C5—C6—C7—C8	2.0 (2)		

### Hydrogen-bond geometry (Å, º)

**Table:**

*D*—H···*A*	*D*—H	H···*A*	*D*···*A*	*D*—H···*A*
O1—H1o···Cl2	0.84 (2)	2.30 (2)	3.1338 (14)	179 (2)
N1—H1n···Cl2^i^	0.88 (1)	2.29 (1)	3.1602 (15)	168 (1)
N2—H2n···Cl2^ii^	0.87 (1)	2.49 (2)	3.2949 (14)	154 (2)
C2—H2···O1^iii^	0.95	2.60	3.545 (2)	173

Symmetry codes: (i) −*x*+2, −*y*+1, −*z*+1; (ii) −*x*+1, −*y*+1, −*z*; (iii) *x*, −*y*+1/2, *z*+1/2.
